# Boundary Cap Neural Crest Stem Cells Promote Survival of Mutant SOD1 Motor Neurons

**DOI:** 10.1007/s13311-016-0505-8

**Published:** 2017-01-09

**Authors:** Tanya Aggarwal, Jan Hoeber, Patrik Ivert, Svitlana Vasylovska, Elena N Kozlova

**Affiliations:** Department of Neuroscience, Uppsala University Biomedical Center, Box 593, 75124 Uppsala, Sweden

**Keywords:** Amyotrophic lateral sclerosis, Neurodegeneration, Neuroglia, Oxidative stress, Transplantation

## Abstract

ALS is a devastating disease resulting in degeneration of motor neurons (MNs) in the brain and spinal cord. The survival of MNs strongly depends on surrounding glial cells and neurotrophic support from muscles. We previously demonstrated that boundary cap neural crest stem cells (bNCSCs) can give rise to neurons and glial cells *in vitro* and *in vivo* and have multiple beneficial effects on co-cultured and co-implanted cells, including neural cells. In this paper, we investigate if bNCSCs may improve survival of MNs harboring a mutant form of human SOD1 (SOD1^G93A^) *in vitro* under normal conditions and oxidative stress and *in vivo* after implantation to the spinal cord. We found that survival of SOD1^G93A^ MNs *in vitro* was increased in the presence of bNCSCs under normal conditions as well as under oxidative stress. In addition, when SOD1^G93A^ MN precursors were implanted to the spinal cord of adult mice, their survival was increased when they were co-implanted with bNCSCs. These findings show that bNCSCs support survival of SOD1^G93A^ MNs in normal conditions and under oxidative stress *in vitro* and improve their survival *in vivo*, suggesting that bNCSCs have a potential for the development of novel stem cell-based therapeutic approaches in ALS models.

**Electronic supplementary material:**

The online version of this article (doi:10.1007/s13311-016-0505-8) contains supplementary material, which is available to authorized users.

## Introduction

Amyotrophic lateral sclerosis (ALS) is a progressive, neurodegenerative disorder affecting primarily upper and lower motor neurons (MNs), and usually leading to the death of the patient, most commonly due to respiratory failure. The glutamate signaling antagonist Riluzole has a measurable positive effect on disease progression and is relatively free of side effects. Riluzole acts by attenuating excitotoxic impact on endangered MNs, and is currently the only available approved ALS treatment, but offers not more than a few months extended life expectancy [1]. Riluzole exerts a wide range of neural effects which can influence neuronal activity and survival, although the mechanism of action still remains controversial [2]. About 10% of ALS cases are familial and several genetic mutations linked to this group of patients have been identified. Mutations in superoxide dismutase (SOD)1 are found in about 20% of familial cases and in a few percent of sporadic ALS cases [3]. SOD1 is an enzyme that helps in the catalysis of dismutation of superoxide to molecular oxygen and hydrogen peroxide [3]. The association between mutant SOD1 and ALS onset, and the subsequent generation of transgenic animals harboring human mutant and non-mutant forms of SOD1, have played a pivotal role and remains a cornerstone in research on the pathogenesis of ALS and in the search for novel therapeutic treatment of this disease [3]. To date, more than 150 SOD1 mutations have been identified and SOD1^G93A^ is the most widely studied model for ALS pathogenesis [3].

Mutant SOD1 affects not only intrinsic properties of MNs, but also results in a neurotoxic astroglial phenotype, inducing degeneration of wild type MNs *in vitro* [4–7] and *in vivo* after transplantation into the spinal cord of rats [8]. Furthermore, astrocytes from patients with sporadic as well as familial ALS exert a toxic effect on primary MNs *in vitro* [9, 10]. Conversely, deletion of mutant SOD1 in astrocytes of transgenic mice significantly delays disease progression [11]. Wild type astrocytes release factors that promote survival of co-cultured mutant SOD1 MNs [12]. Implantation of stem/progenitor cell-derived astrocytes into the spinal cord or ventricular system of transgenic mice with mutant SOD1 promotes MN survival and delays disease progression [13, 14].

Boundary cap neural crest stem cells (bNCSCs) is a transient neural crest-derived group of cells that are located at the dorsal root entry zone (DREZ) [15]. These cells self-renew and show multipotency in culture and are able to differentiate into sensory neurons and Schwann cells *in vitro* and *in vivo* [15], as well as into astrocytes *in vitro* and after transplantation into the immature mouse brain [16]. We have previously shown remarkable, beneficial effects of bNCSCs on co-cultured [17, 18] and co-implanted pancreatic beta-cells [19], as well as excitotoxically challenged spinal cord neurons *in vitro* (unpublished observation). Interestingly, another type of NCSCs, the hair follicle stem cells, did not have corresponding positive effects on co-cultured cells [20].

These findings prompted us to test if bNCSCs have a beneficial effect on co-cultured and co-implanted SOD1^G93A^MNs, generated from SOD1^G93A^ mouse embryonic stem cells (mESCs). These cell lines express green fluorescent protein (GFP) under the control of the promoter for the MN specific transcription factor HB9 (*HB9*::GFP cells), allowing their identification after directed differentiation to MN progenitors [21]. Furthermore, the survival of MN from the same SOD1^G93A^ mESC line was previously analyzed *in vitro* under normal conditions [21]. Here, we investigate their survival in normal conditions and under oxidative stress and the effect of bNCSCs on SOD1^G93A^ MN survival. Generation of MNs from mESCs results also in abundant generation of astrocytes. These cells express glutamate aspartate transporter (GLAST) and can be identified by anti-GLAST antibodies [22]. To exclude the negative effect from surrounding SOD1^G93A^ astrocytes on SOD1^G93A^ MNs, we used magnetic activated cell sorting (MACS) to eliminate GLAST-positive cells from SOD1^G93A^ mESC cultures. To compare the effect of bNCSCs on SOD1^G93A^ MN survival with mESC derived astrocytes, we used astrocytes differentiated from a non-SOD1 mutated glial fibrillary acidic protein (*GFAP)*::CD14 mESC line [23].

Our findings show that bNCSCs exert a significant survival promoting effect on co-cultured and co-implanted SOD1^G93A^ MNs.

## Material and methods

### Mouse embryonic stem cell (mESC) cultures

Mouse embryonic stem cell (mESC) lines harboring human wild type SOD1 (SOD1^WT^) or mutant SOD1 (SOD1^G93A^) were a kind gift from Dr. Kevin Eggan (Harvard Stem Cell Institute). The risk of tumor formation for implanted cells from low passages is minimal. These cell lines carry green fluorescent protein (GFP) under the control of the promoter for the MN specific transcription factor HB9 (*HB9*::GFP cells) [21]. We used the SOD1^WT^ and SOD1^G93A^ mESC lines to derive GFP^+^ MNs.

For MN differentiation, a previously published protocol with small modifications was used [24]. Cells were cultured in ADFNB medium consisting of Advanced D-MEM/F12:Neurobasal (1:1), 1x GlutaMAX supplement (Invitrogen), 1x B27 supplement (Invitrogen), 1x N2 supplement (Invitrogen), 0.1 mM 2-mercaptoethanol (Sigma) to form embryoid bodies (EBs), and supplemented with 0.1 μM of retinoic acid (RA, Sigma) and 0.5 μM of sonic hedgehog (Shh) agonist Ag1.3 (Curis) every other day. After 7 days of pre-differentiation, EBs were subjected to MACS (see below), and thereafter either cultured alone, co-cultured with bNCSCs, or with mESC-derived astrocytes.

For *in vitro* MN differentiation, EBs were enzymatically dissociated with TrypLE™ Express (Gibco) and seeded on pre-coated coverslips with 0.01% poly-l-ornithine (Sigma) followed by 10 μg/mL laminin (Sigma). Cells were seeded at a density of 5 × 10^4^ cells/coverslip in 24 well plates with ADFNB cell medium supplemented with 10 ng/mL of CNTF (Miltenyi Biotec) and GDNF (Miltenyi Biotec). 50% of the medium was replaced with fresh medium every other day until the cultures were fixed in 4% paraformaldehyde in phosphate buffered saline (PBS; 137 mM NaCl, 2.7 mM KCl, 100 mM Na_2_HPO_4_, 18 mM KH_2_PO_4_) at the indicated time points.

### bNCSC culture

bNCSCs were generated from transgenic mice harboring red fluorescent protein (RFP) under the universal actin promoter [25] according to previously published protocols [15, 26]. Neurospheres from passages 4 to 5 were trypsinized to obtain single cell suspensions for MACS for subsequent co-culture and co-implantation with SOD1^G93A^ MNs.

### Derivation of astrocytes from *GFAP*::CD14 mESC culture

Astrocytes were generated from *GFAP*::CD14 mESCs (kind gift from Dr. Ivo Lieberam, Kings College, London), using MACS as has been previously described [23] with minor modifications. At day 7 of differentiation, the EBs were re-plated into T75 flasks (Sarstedt) pre-coated with 10 μg/mL laminin and cultured until day 12 in ADFNB medium. The monolayer formed by EBs was treated with TrypLE™ Express on day 12 and a single cell suspension was prepared for MACS. Anti-CD14 antibody conjugated with magnetic microbeads was used according to the manufacturers protocol (Miltenyi Biotec).

### Magnetic activated cell sorting (MACS)

Anti-GLAST (ACSA-1) antibodies were used for MACS according to the manufacturers protocol (Miltenyi Biotec). bNCSCs and *GFAP*::CD14 EBs were subjected to MACS to obtain astrocyte cultures. SOD1^G93A^ EBs were subjected to MACS using anti-GLAST to deplete SOD1^G93A^ MN cultures from SOD1^G93A^ astrocytes.

Culture suspensions containing around 3 × 10^6^ cells were labeled with anti-GLAST antibodies. Thereafter, anti-mouse IgG magnetic microbeads (Miltenyi Biotec) were applied to the cells for 15 minutes at 4 °C. Cells were re-suspended in MACS buffer (Miltenyi Biotec) and the cell suspension was loaded onto a MACS separation column (Miltenyi Biotec) and placed in the magnetic field of a MACS separator.

The column was rinsed three times with 0.5 mL of MACS buffer. In the case of the SOD1^G93A^ cell line, the GLAST-positive fraction was removed to minimize the influence of SOD1^G93A^ astrocytes on SOD1^G93A^ MNs. For bNCSCs and *GFAP*::CD14 EBs, the GLAST positive fractions were used for the experiments.

For differentiation assays, 5 × 10^4^ of SOD1^G93A^ cells were seeded on 0.01% poly-l-ornithine and 10 μg/mL laminin coated coverslips. In case of co-cultures, around 2 x 10^4^ bNCSCs or *GFAP::*CD14 mESC-derived astrocytes were seeded first followed by SOD1^G93A^ MNs.

### Oxidative stress assay

Oxidative stress was induced by hydrogen peroxide (H_2_O_2_) in SOD1^WT^ MNs and SOD1^G93A^ MNs plated alone or together with bNCSCs. In addition, bNCSCs were treated alone with H_2_O_2_ at similar concentrations to test their survival capacity under these conditions. On day 4 of differentiation assay, H_2_O_2_ (60 μM, 150 μM, 300 μM) was added for 3 hours to the cultures. Thereafter, the coverslips were fixed and analyzed for MN or bNCSC survival (GFP-positive or RFP-positive cells, respectively). For each condition at least three experiments were performed.

### Animals

Transplantations were performed in Crl:NU(NCr)-Foxn1nu (nude; nu/nu) NMRI adult male mice (n = 12), (body weight 25–35 g; Möllegaard and Bomholgard Breeding and Research Centre, M&B A/S, Bomholt, Denmark, http://www.taconic.com). The serial sections were from the spinal cords of six animals − three of which received bNCSCs and SOD1^G93A^ MN precursors (treatment group) and three only SOD1^G93A^ MN precursors (control group) − were analyzed under confocal microscope for MN survival and glial response. All animal experiments were approved by the Local Ethical Committee for Animal Experimentation, Uppsala, as required by Swedish Legislation and in accordance with European Union Directives.

### Surgery

The animals were anesthetized by spontaneous inhalation of Isoflurane. After onset of anesthesia the mouse was placed on its stomach on a heating pad (36 °C) and the skin cleaned with ethanol (70%). Following a procedure previously established in rats [27], an incision was made in the midline of the neck skin and local anesthetic (Xylocain©, 10 mg/ml) applied to the wound. After blunt dissection of the superficial and deep back muscles, the laminae of the cervical vertebrae were exposed. The vertebra prominens (C7) was held fixed with a pair of forceps, a partial laminectomy was made of vertebrae C3 to C5, and the exposed meninges were gently opened. 4 μL (2 injections of 2 μL each, around 50 000 cells per mouse) of equal proportion of bNCSCs and SOD1^G93A^ MN precursors were injected into the left spinal cord using a Hamilton syringe with a metal needle (26 gauge) attached to a stereotactic frame and connected to an infusion pump (KD Scientific Legato 130). For the control group an equal amount of cells containing only SOD1^G93A^ MN precursors were injected. The needle was kept in place for 2 minutes before removal. The wound was closed in layers using ethilon nylon suture 6.0 and 4.0, respectively. The animals were given 50 μl buprenorphine (Temgesic©) subcutaneously every 12 hours for 3 days after the operation.

Seven days after surgery the animals were re-anesthetized with an intraperitoneal injection of a mixture of ketamine, xylazine and acepromazine [28], and perfused via the left ventricle with warm saline solution (~38 °C) followed by a cold (~4 °C) fixative solution consisting of 4% formaldehyde (vol/vol), 14% saturated picric acid (wt/vol) in phosphate buffered saline (PBS; pH 7.35-7.45). The relevant part of the cervical spinal cord was removed, placed in fixative solution for 4 hours, and thereafter cryoprotected overnight in PBS containing 15% sucrose. The following day the tissue was placed in TissueTech™ and frozen in liquid nitrogen. Transverse sections (14 μm) were cut on a cryostat, collected on SuperFrost™ Plus slides (Menzel-Gläser, Braunschweig, Germany), and processed for immunohistochemistry and microscopic analysis as described below.

### Immunohistochemistry

Cells were fixed in 4% phosphate-buffered paraformaldehyde at room temperature (RT) for 5 minutes, washed and left in blocking solution (1% bovine serum albumin, 0.3% Triton X-100 and 0.1% NaN_3_ in PBS) for 60 minutes, and then incubated with primary antibodies overnight at 4 °C, followed by the appropriate secondary antibodies for 1 hour at RT (Table 1). During the final wash, the cells were incubated with Hoechst 33342 (1:10000; Invitrogen) to label cell nuclei, and then mounted on a glass slide for analysis. bNCSC neurospheres were fixed for 30 minutes, immersed in 15% sucrose overnight, cryosectioned the next day and sections processed for immunohistochemistry.

**Table 1:** Antibodies used in the study

Antigen	Host	Catalog number	Source	Dilution
*Primary*				
Human SOD1 misfolded	Mouse	MM-0070-2	Medimabs	1:100
GFAP	Rabbit	2016-04	Dako	1:500
Iba1	Rabbit	019-19741	Wako	1:500
Beta-3-tubulin	Mouse	32-2600	Invitrogen	1:500
ACSA-1 (GLAST)	Mouse	130-095-822	Miltenyi Biotec	1:100
SOX2	Goat	Sc-17320	Santa Cruz	1:200
GFP-FITC	Goat	ab6662	Abcam	1:250
*Secondary*				
Alexa 647	Donkey α mouse	A31571	Invitrogen	1:500
Alexa 647	Donkey α rabbit	A31573	Invitrogen	1:500
FITC	Donkey α goat	705-095-147	Jackson ImmunoResearch	1:200
AMCA 350	Donkey α rabbit	711-155-152	Jackson ImmunoResearch	1:100
Cy3	Donkey α mouse	715-165-151	Jackson ImmunoResearch	1:500

Transverse sections from the C3-5 spinal cord were pre-incubated with blocking solution for 1 hr at RT and then incubated overnight at 4 °C with primary antibodies for the astroglial marker GFAP or microglia/macrophage marker Iba1 followed by the appropriate secondary antibodies and anti-GFP-FITC for 1 hour at RT (Table 1).

### Microscopy and cell counting

Cells on coverslips and spinal cord cryosections were captured using a 20× objective (NA 0.75) of a Nikon Eclipse E800 epifluorescence microscope equipped with a Nikon DXM1200F CCD camera. For cultured cells, GFP and RFP positive cells were counted manually for each condition. GFP positive cells were counted in every 5th cryosection along the length of the transplant area (ranging from ~0.8 to 1.5 mm along the cranial-caudal axis between animals). Sections were analysed using a Zeiss LSM700 or a Zeiss LSM780 confocal laser scanning microscope. Images were captured using a LD LCI Plan-Apochromat 20x (NA 0.8) or a LD C-Apochromat 40x W Corr (NA 1.1) objective.

### Statistical Analysis

Datasets for oxidative stress and survival assays were analyzed using two-way ANOVA followed by the Bonferroni multiple comparisons tests. MN survival was analyzed using a two-tailed Students t-test. All statistical analyses were performed in GraphPad Prism 5.04. The confidence interval was stated at the 95% confidence level, placing statistical significance at p < 0.05.

## Results

### Improved survival of SOD1^G93A^ MNs *in vitro*

Both SOD1^WT^ and SOD1^G93A^ mESC lines formed EBs with abundant expression of *HB9::*GFP and gave rise to GFP^+^ MN precursors in culture (Supplementary Fig. 1).

bNCSCs neurospheres abundantly expressed RFP, the neural crest stem cell marker SOX2 and GLAST (after MACS purification), and continued to express RFP in differentiation assay in co-culture and co-implants (Supplementary Figs. 2, 3 and 4).

**Fig. 1:**
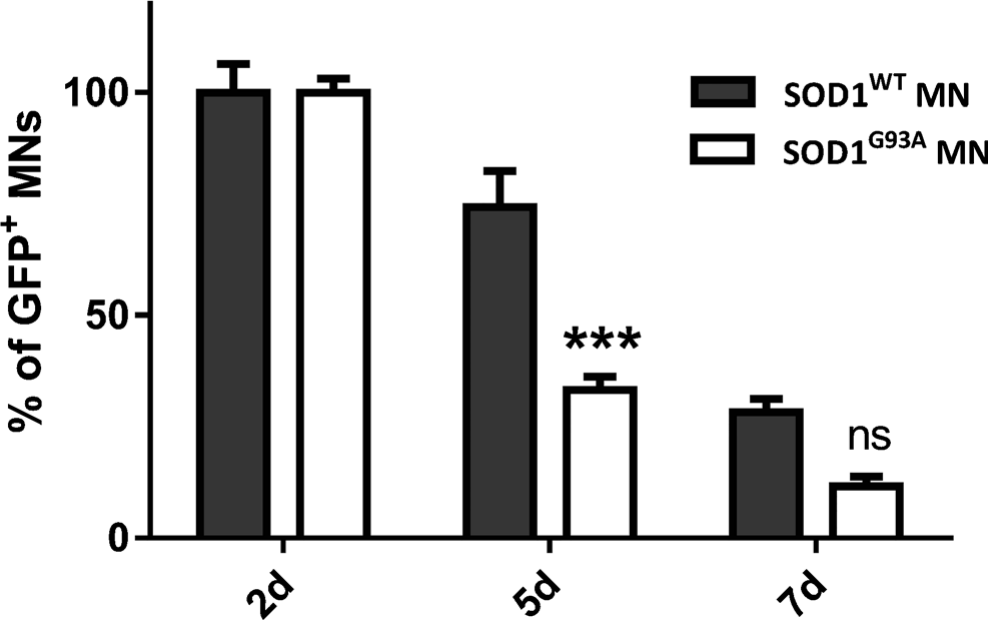
Survival of SOD1^WT^ and SOD1^G93A^ MNs *in vitro.* The SOD1^G93A^ cell line shows a reduced MN survival compared to the SOD1^WT^ cell line between days 2 and 7. *Asterisks* indicate the level of statistical significance by two-way ANOVA followed by Bonferroni multiple comparison test (*** p < 0.001). Data shown is in mean ± SEM of three independent experiments

The poor survival of SOD1^G93A^ MNs *in vitro* has been compared to MNs derived from the *HB9*::GFP cell line in a previous study [21]. Here we compare the survival of SOD1^G93A^ MNs with SOD1^WT^ MNs to confirm that the reduced survival is due to the SOD1^G93A^ mutation. Cells from both cultures were subjected to differentiation assay and GFP^+^ MNs were counted after 2, 5 and 7 days of differentiation. The number of MNs declined more rapidly in SOD1^G93A^ cultures at day 5 of differentiation (p < 0.001) (Fig. 1).

During MN differentiation from mESCs, a population of astrocytes is also present. Previous studies have shown a negative effect of SOD1^G93A^ astrocytes on MNs [21]. We therefore examined if a reduction of the astrocyte population in SOD1^G93A^ cultures will improve MN survival and if co-culture with bNCSCs, or with *GFAP*::CD14 astrocytes will improve the survival of SOD1^G93A^ MNs.

**Fig. 2:**
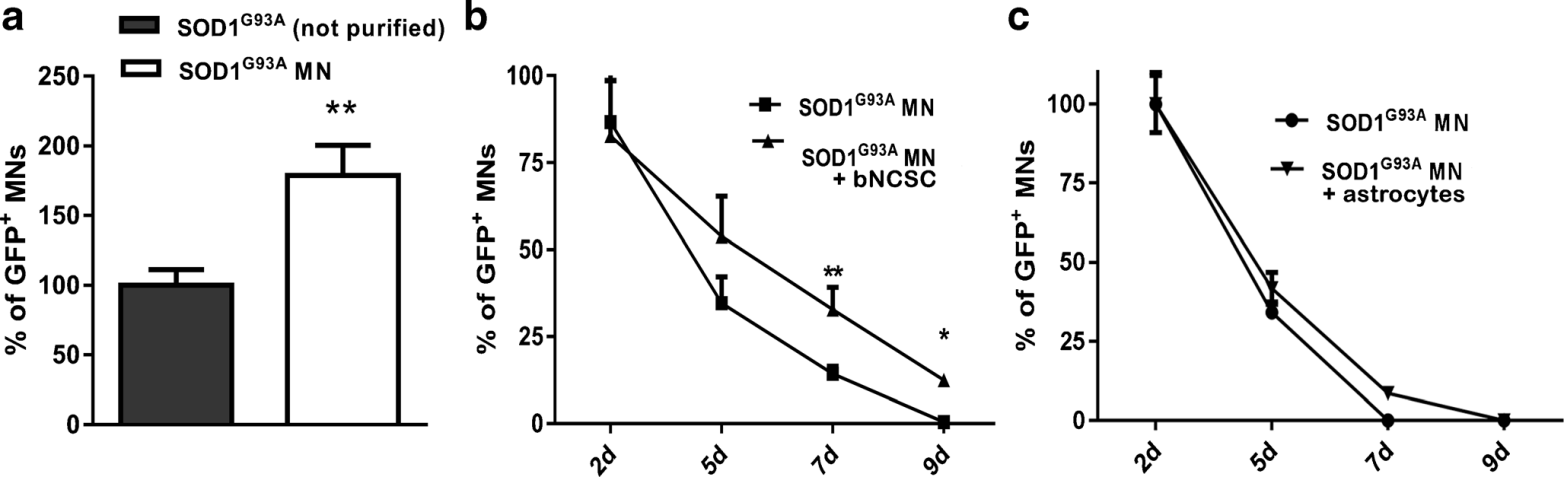
Increased survival of SOD1^G93A^ MNs *in vitro* by removal of SOD1^G93A^ astrocytes and addition of bNCSC. Removal of astrocytes from SOD1^G93A^ cell cultures results in an increased number of SOD1^G93A^ MNs **(a)**. Survival of SOD1^G93A^ MNs increased when co-cultured with bNCSCs **(b)**. SOD1^G93A^ MN survival showed no improvement when co-cultured with *GFAP*::CD14 astrocytes **(c)**. Asterisks indicate the level of statistical significance by two-tailed Student’s test **(a)** or two-way ANOVA **(b)** (** p < 0.01,*p < 0.05). Data shown is in mean ± SEM of three independent experiments

After reduction of the astrocyte population in SOD1^G93A^ cultures there was an approximate 75% increase in the number of SOD1^G93A^ MNs 12 hours after purification compared to non-purified cell cultures (Fig. 2a; p < 0.01). The survival of SOD1^G93A^ MNs was significantly increased in co-culture with bNCSC by day 7 (Fig. 2b; p < 0.01 and p < 0.05), whereas co-culture of SOD1^G93A^ MNs with *GFAP*::CD14 astrocytes did not affect MN survival (Fig. 2c).

**Fig. 3:**
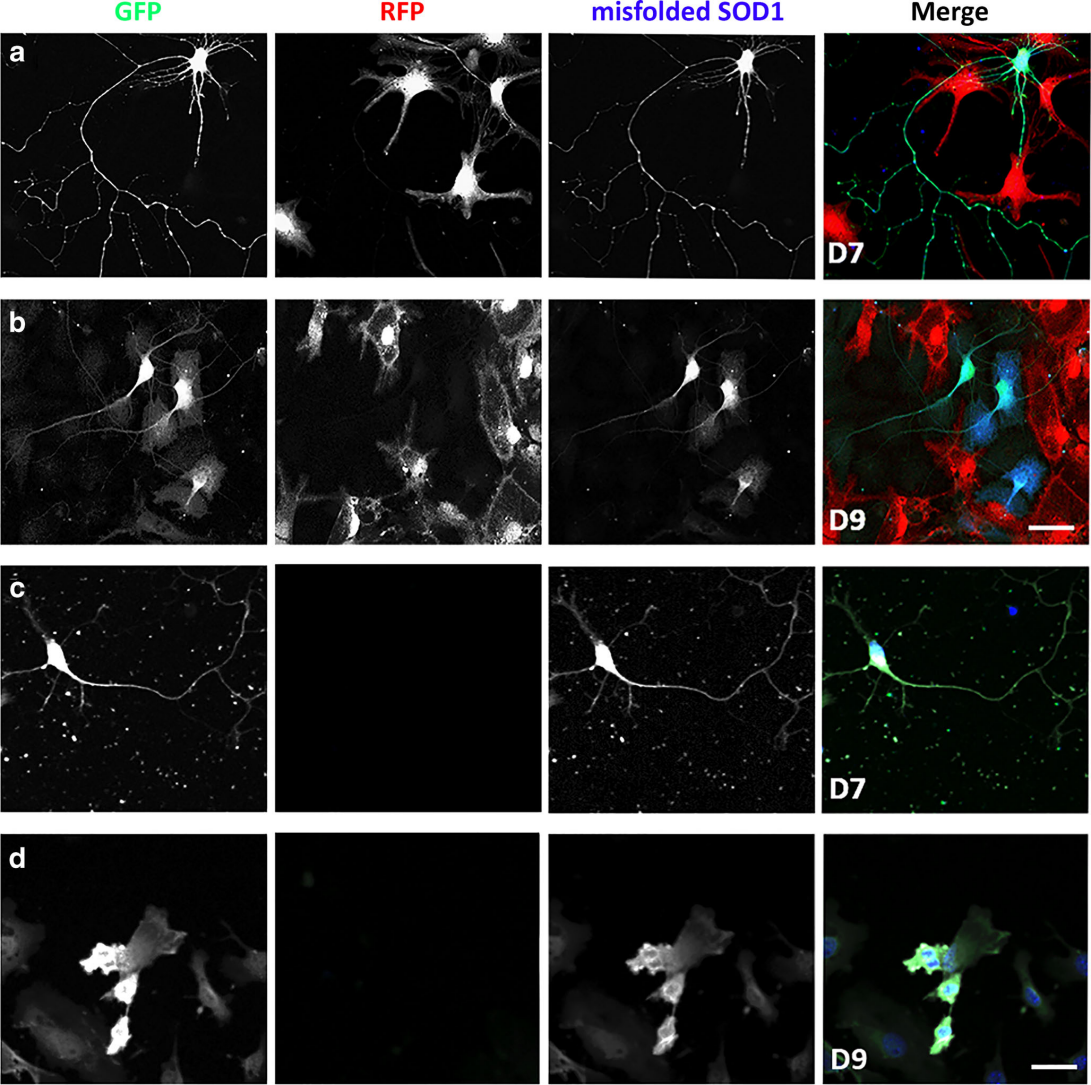
SOD1^G93A^ MNs in the presence or absence of bNCSCs *in vitro.* Co-cultured SOD1^G93A^ cells (GFP) and bNCSCs (RFP) display close contact on day 7 **(a)** and 9 **(b)**, misfolded SOD1 was only detected in SOD1^G93A^ MNs. SOD1^G93A^ MNs cultured alone show reduced survival and the presence of misfolded SOD1 on day 7 **(c)** and day 9 **(d)**. Scale bar: 50 μm

We detected close contact between SOD1^G93A^ MNs and bNCSCs, but did not observe immunoreactivity for misfolded SOD1 in bNCSCs, suggesting that aggregated SOD1 was not transferred from SOD1^G93A^ MNs to adjacent bNCSCs (Fig. 3).

### bNCSCs improve survival of SOD1^G93A^ MNs *in vitro* under oxidative stress

**Fig. 4:**
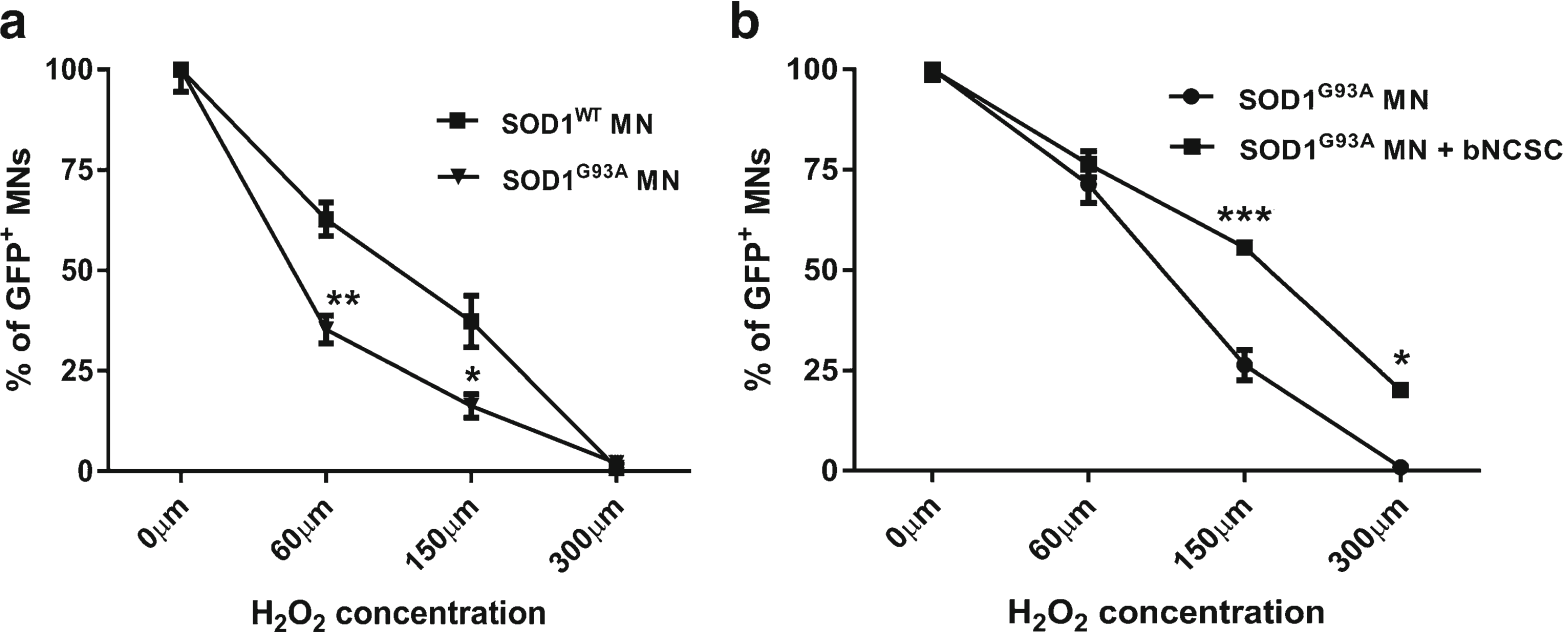
Effect of hydrogen peroxide (H_2_O_2_) on SOD1^G93A^ MN survival *in vitro.* Survival of SOD1^G93A^ MNs was reduced compared to SOD1^WT^ MNs under oxidative stress **(a)**. Survival of SOD1^G93A^ MNs increased when co-cultured with bNCSCs (SOD1^G93A^ MNs + bNCSCs) compared to when cultured alone (SOD1^G93A^ MN) **(b)**. *Asterisks* indicate level of statistical significance by two-way ANOVA followed by Bonferroni multiple comparison test (*** p < 0.001, ** p < 0.01, *p < 0.05). Data shown is in mean ± SEM of three independent experiments

We compared the survival of SOD1^G93A^ MNs and SOD1^WT^ MNs under oxidative stress. Survival of SOD1^G93A^ MNs was markedly decreased (p < 0.01 and p < 0.05) compared to SOD1^WT^ MNs, indicating that SOD1^G93A^ MNs are more susceptible than wildtype MNs to oxidative stress (Fig. 4a). Co-culture of SOD1^G93A^ MNs with bNCSCs under oxidative stress showed a significant increase in SOD1^G93A^ MN survival compared to SOD1^G93A^ MNs cultured alone (p < 0.001 and p < 0.05, Fig. 4b).

We next tested if bNCSCs are susceptible to oxidative stress under similar oxidative stress conditions and found no effect of H_2_O_2_ exposure on the survival of bNCSCs *in vitro* (Supplementary Fig. 3).

### SOD1^G93A^ MNs show increased survival in the presence of bNCSCs *in vivo*

**Fig. 5:**
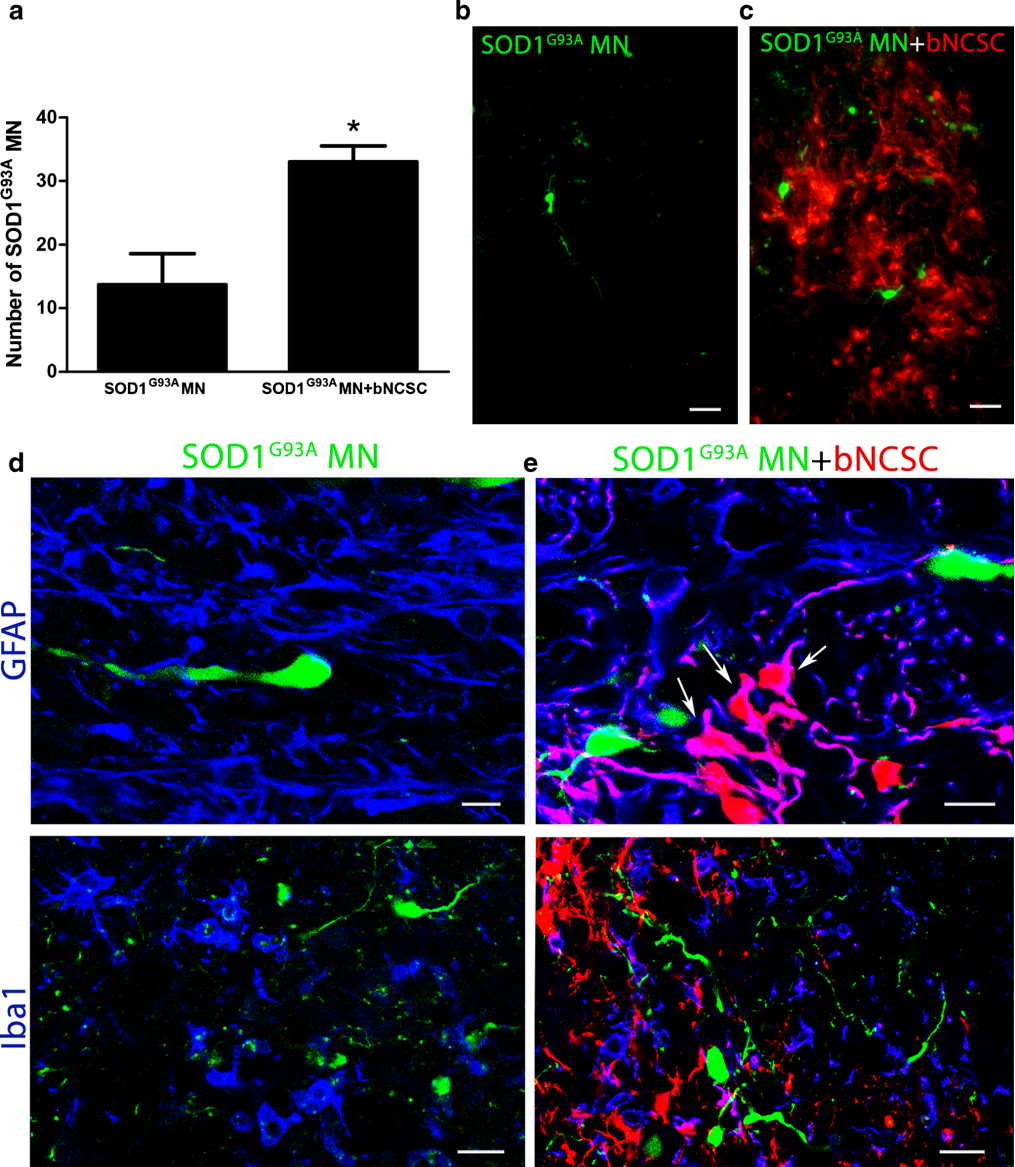
Increased survival of SOD1^G93A^ MN after co-implantation with bNCSC to the spinal cord of mice. One week after implantation, SOD1^G93A^ MN co-implanted with bNCSC showed significantly increased survival compared to SOD1^G93A^ MN implanted alone. *Asterisks* indicate the level of statistical significance by two-tailed Student’s t-test (* p < 0.05). Data shown is in mean ± SEM of three animals per condition **(a)**. SOD1^G93A^ MNs when implanted alone are located as single cells in the spinal cord parenchyma **(b)**. When implanted together with bNCSCs, SOD1^G93A^ MNs can be found both spread out as single cells as well as in close proximity to bNCSCs **(c)**. The injection site showed increased immunoreactivity for GFAP and Iba1 following implantation of either SOD1G93A MN alone **(d)** or co-implantation with bNCSCs **(e)**. *Arrows* indicate bNCSCs differentiated to GFAP positive cells **(e)**. Scale bars: b,c 25 μm; d,e 10 μm for GFAP and 20 μm for Iba1

SOD1^G93A^ MNs alone or together with bNCSCs were implanted into the left spinal cord of nude mice, and the survival of implanted MNs was assessed one week after implantation. In all animals which received SOD1^G93A^ MN precursors together with bNCSCs, significantly more GFP^+^ SOD1^G93A^ MNs were detected compared to animals that received SOD1^G93A^ MN precursors alone (Student’s t-test, p < 0.05, Fig. 5a). The surviving MNs were located in the proximity of bNCSCs or localized as single cells in the spinal cord parenchyma (Fig. 5b,c). Immunolabeling with the astrocytic marker GFAP revealed that bNCSCs differentiated primarily to GFAP positive cells (Fig. 5e).

**Fig. 6:**
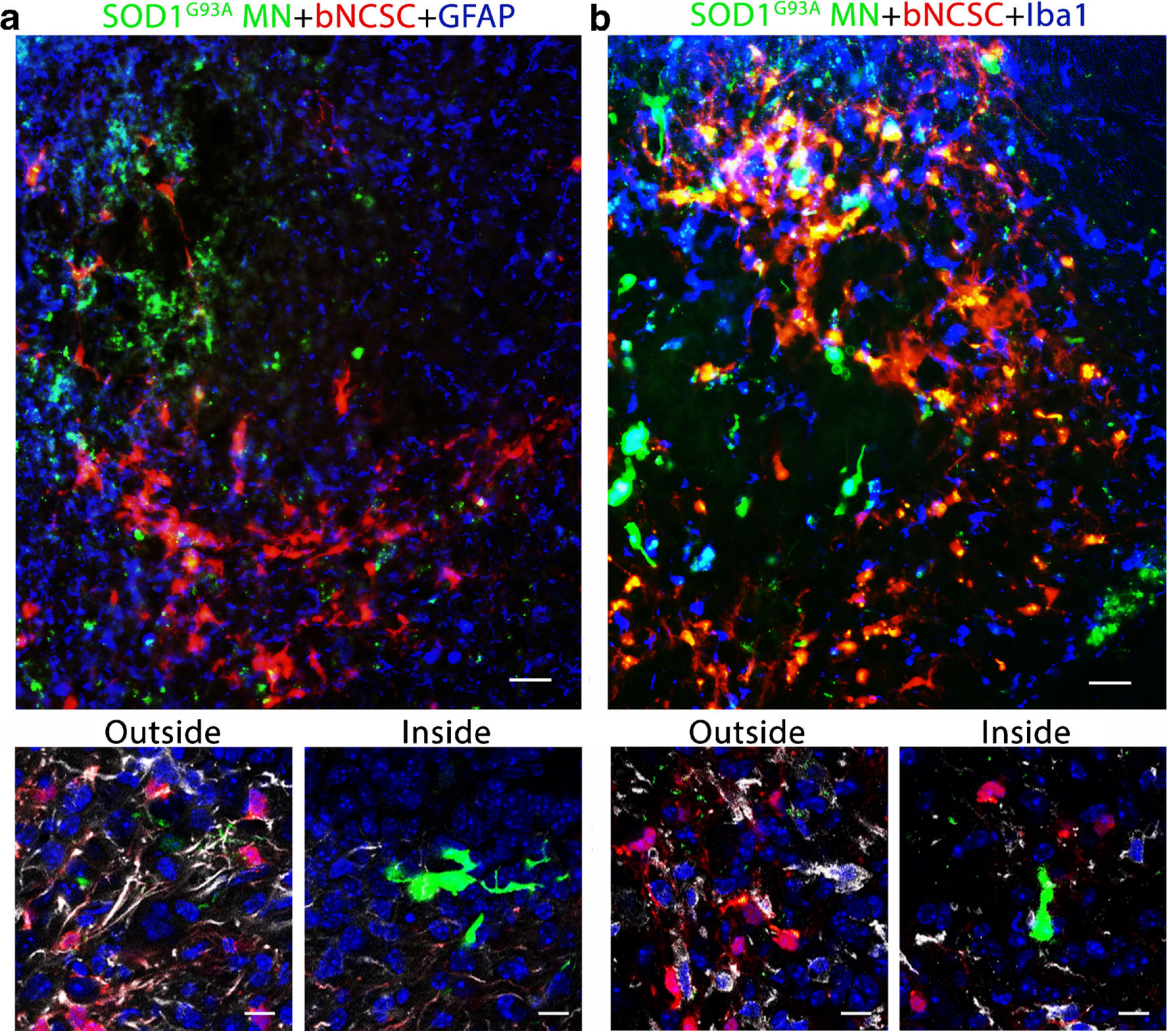
Transplanted bNCSCs might diminish the local glial response following implantation. Spinal cord areas partially surrounded by bNCSC transplants showed decreased immunoreactivity for GFAP **(a,** *inside* **)** and Iba1 **(b,** *inside* **)** and a high number of co-implanted SOD1^G93A^ MN **(a, b)**. Overviews show GFAP and Iba1 in *blue*, high magnification confocal images show GFAP and Iba1 in *white* and cell nuclei in *blue* for areas inside and outside the area of bNCSC transplantation. Scale bars: a,b 25 μm for overviews and 10 μm for high magnification images

The improved survival of MNs in the presence of bNCSCs after implantation prompted us to investigate if bNCSCs affect the glial response in the recipient spinal cord in the vicinity of implanted MN precursors. As expected, there was an increased immunolabeling for GFAP and Iba1 from the minor injury at the injection site, but these changes were similar in the two experimental groups (Fig. 5d,e). However. the glial response for GFAP and Iba1 within the area where bNCSCs were located seemed reduced compared to adjacent areas lacking bNCSCs (Fig. 6a,b).

## Discussion

There is an urgent need for novel therapeutic strategies with enhanced effect for ALS patients. Here we show that bNCSCs exert a significant survival promoting effect on SOD1^G93A^ MN *in vitro* as well as after co-implantation to the spinal cord. Previously, we detected several beneficial effects of bNCSCs on co-cultured and co-transplanted cells. Thus, bNCSCs strongly increase proliferation of insulin producing beta-cells in co-transplants of mouse pancreatic islets in streptozotocin diabetic mice [19, 29], and in co-culture with beta-cells [17]. bNCSCs protect insulin-producing cells from cytokine induced apoptosis *in vitro* [18], an effect which appears to require direct bNCSC- cell contact through catenin-cadherin junctions [30]. Co-implantation of bNCSCs with mouse and human pancreatic islets improves their survival after transplantation and increases beta-cell proliferation, vascularization of transplants and their re-innervation [29]. We also found their survival supporting effect in spinal cord slice cultures exposed to excitotoxic stress (unpublished observation).

The conditions for implanting healthy supportive cells into the spinal cord of ALS patients imply that these cells will have not only to survive in the harmful ALS environment, but also exert beneficial effects on diseased MNs. For this reason we have tested if bNCSCs are susceptible to oxidative stress and found that they are resistant to exposure to hydrogen peroxide in concentrations which significantly impair survival of SOD1^G93A^ MNs. We next examined whether bNCSCs are “contaminated” with misfolded SOD1 from co-cultured SOD1^G93A^ MNs. ALS appears to begin focally at a random location and progresses contiguously through two distinct types of neuroanatomic propagation: contiguous propagation through the extracellular matrix independent of synaptic connections, and network propagation, which is dependent on synaptic connections [31]. In many neurodegenerative disorders misfolded proteins appear to contribute to disease progression [32]. Thus, the presence of misfolded SOD1 may contribute to disease propagation in some forms of ALS [33]. We detected immunoreactivity for misfolded SOD1 in SOD1^G93A^ MNs generated from SOD1^G93A^ mESCs. Although astrocytes co-cultured with SOD1^G93A^ MNs have been reported to be "contaminated" with aggregated SOD1 [34], we did not detect aggregated SOD1 in bNCSCs, suggesting that they are resistant to relocation of misfolded SOD1. Thus, bNCSCs display properties *in vitro* that may be useful for disease modifying cell therapy in ALS.

We explored these properties in co-cultures of bNCSCs with SOD1^G93A^ MNs subjected to oxidative stress, and demonstrated significantly increased SOD1^G93A^ MN survival compared to untreated SOD1^G93A^ MN cultures. In agreement with previous studies [11], we found that depletion of the astrocyte population from SOD1^G93A^ cultures, increases MN survival. The addition of bNCSCs, but not healthy astrocytes, to these cultures further improved the survival of SOD1^G93A^ MNs under both normal and oxidative stress conditions. This finding provides additional evidence for the potency of bNCSCs in supporting diseased SOD1^G93A^ MNs. The improved SOD1^G93A^ MN survival coincided with the presence of direct contacts between bNCSCs and SOD1^G93A^ MNs, suggesting that the positive effect of bNCSCs might be mediated through intercellular connections, as was shown previously in bNCSC-beta-cell cultures [29]. We have also shown that bNCSCs produce a broad range of trophic factors, as well as Wnt-1 and matrix metalloproteinases [35]. This trophic capacity, in co-operation with direct cell-cell interaction, may explain the survival promoting effects of bNCSCs on SOD1^G93A^ MNs under stressful conditions *in vitro*.

Pre-differentiated neural cells are particularly vulnerable during the initial period after implantation into the central nervous system. At this stage environmental conditions that support survival and differentiation of implanted cells are likely to be critical for their subsequent integration into neural circuits and long-term survival. We therefore focused on the viability of SOD1^G93A^ MNs alone, or in combination with bNCSCs, during the first week of their implantation. One week after implantation of SOD1^G93A^ MN precursors to the spinal cord, only few surviving SOD1^G93A^ MNs were found. Survival of SOD1^G93A^ MNs was, however, significantly increased when co-implanted with bNCSCs, despite the fact that twice as many SOD1^G93A^ MN precursors were implanted alone compared to the number of SOD1^G93A^ MN precursors co-implanted with bNCSCs. These findings show that bNCSCs create environmental conditions for SOD1^G93A^ MNs, which support their survival in the spinal cord.

Inflammatory components have been implicated in the neurodegeneration associated with ALS [36]. As expected, the injection procedure resulted in a local microglial and astroglial response at the site of cell implantation, but this response did not differ between the two experimental conditions. However, when areas harboring implanted cells were analyzed, immunoreactivity for host microglia/macrophages and astrocytes appeared reduced where SOD1^G93A^ and bNCSCs were co-localized, suggesting that bNCSCs are able to attenuate the local inflammatory response associated with implanted SOD1^G93A^ MNs. The survival support by bNCSCs may initially rely on direct cell-cell interactions at the site of cell implantation where bNCSCs and SOD1^G93A^ MN precursors intermingle during the injection procedure. However, the presence of GFP^+^ MNs at a distance from bNCSCs and in the vicinity of the central canal one week after implantation indicates that some implanted SOD1^G93A^ MN precursors rapidly become independent of close contact with bNCSCs. Survival of migrated SOD1^G93A^ MNs may be supported by bNCSC-derived diffusible molecules, such as vascular endothelial growth factor (VEGF), brain-derived neurotrophic factor (BDNF), glial-derived neurotrophic factor (GDNF), and ciliary neurotrophic factor (CNTF) [35], all of which have trophic effects on MNs [37]. bNCSC-derived matrix metalloproteinase-2 and -9 (MMP-2 and -9) [36], which have been implicated in cell migration [38, 39], may contribute to the migratory capacity of implanted SOD1^G93A^ MNs to the spinal cord parenchyma and in some cases towards the central canal (Supplemental Fig. 4). The ependyma which surrounds this canal is a source of growth factors [40] and was previously designated as a stem cell region in the adult spinal cord [41]. Previous studies have shown that stem cells readily generate neurons when transplanted into neurogenic areas of the adult brain [42, 43]. The survival of SOD1^G93A^ MN precursors in the central canal area suggests that this is a region in the spinal cord with neurogenic properties, which are beneficial for the survival of implanted MN precursors.

The ability of bNCSCs to promote vascularization of co-implanted tissue [29] may also have contributed to improved survival of co-implanted SOD1^G93A^ MNs. Previous studies have shown that ALS is associated with disruption of the blood-CNS-barrier (B-CNS-B) [44–46], a process that can contribute to the inflammatory response in areas affected by ALS. An interesting aspect in this context is whether the angiogenetic properties of bNCSCs in combination with their preferential post-implantation differentiation to healthy SOD1^G93A^ resistant astrocytes may contribute to the formation of new blood vessels with an intact B-CNS-B in the diseased spinal cord. Implantation of bNCSCs to animal models of ALS may clarify the potential of these cells to recover B-CNS-B.

In conclusion, we show that bNCSCs promote survival of SOD1^G93A^ MNs *in vitro* and *in vivo* after co-implantation to the spinal cord. The beneficial effects by bNCSCs might be due to their resistance to oxidative stress and their neurotrophic and angiogenic support. These properties in combination with their resistance to SOD1^G93A^ aggregates make them interesting candidates for further investigations on novel therapeutic approaches to achieve long-term neuroprotection in animal models of ALS.

## Electronic supplementary material

Below is the link to the electronic supplementary material.

Supplementary Fig. 1 SOD1^WT^ and SOD1^G93A^ EBs abundantly expressed *HB9::*GFP in MN precursors on day 7 **(a, b)** and generated GFP^+^ MNs *in vitro* **(c, d),** expressing Beta-3-tubulin **(e, f, g, h)**. Scale bar: 50 μm **(d)**, 20 μm **(h) (GIF 137 kb)**

High Resolution Image (TIF 3969 kb)

Supplementary Fig. 2 Overview of RFP-expressing bNCSC neurospheres in pre-differentiation **(a)** and the bNCSC monolayer during differentiation stage **(b)**. Immunostaining of bNCSC neurospheres shows expression of the glial specific marker GLAST **(c)** and the neural crest stem cell specific markers SOX2 **(d)**. Scale bars: a,b 20 μm; c,d 50 μm. (GIF 164 kb)

High Resolution Image (TIF 6408 kb)

Supplementary Fig. 3 Effect of hydrogen peroxide (H_2_O_2_) on the survival of bNCSCs *in vitro*. Cultures were exposed to different concentrations of H_2_O_2_ for 3 hours. The survival of bNCSCs is not affected by different concentrations of H_2_O_2 (GIF 5 kb)_

High Resolution Image (TIF 18434 kb)

Supplementary Fig. 4 bNCSCs and SOD1^G93A^ MNs one week after implantation to the ventral horn. SOD1^G93A^ MNs (*green*) are located in the grey matter close to the central canal and together with bNCSCs (*red*) in the white matter of the ventral funiculus. Scale bar: 50 μm (GIF 127 kb)

High Resolution Image (TIF 3407 kb)

ESM 1 (PDF 1224 kb)
