# Supplementary figures and images for: Boundary Cap Neural Crest Stem Cells Promote Survival of Mutant SOD1 Motor Neurons
Source: Neurotherapeutics. 2017 Jan 9;14(3):773–83. doi: 10.1007/s13311-016-0505-8 (PMC5509618; doi:10.1007/s13311-016-0505-8)

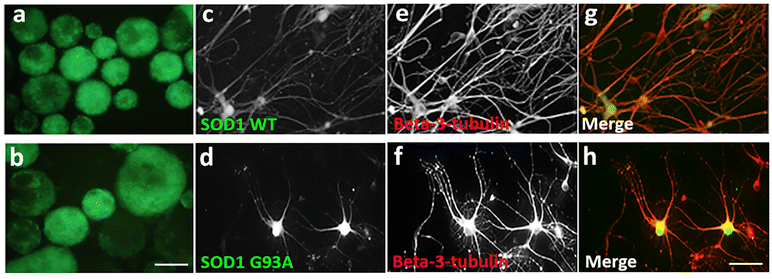

Supplement: Supplementary file 1 — SOD1WT and SOD1G93A EBs abundantly expressed HB9::GFP in MN precursors on day 7 (a, b) and generated GFP+ MNs in vitro (c, d), expressing Beta-3-tubulin (e, f, g, h). Scale bar: 50 μm (d), 20 μm (h) (GIF 137 kb) [file 13311_2016_505_Fig7_ESM.gif]

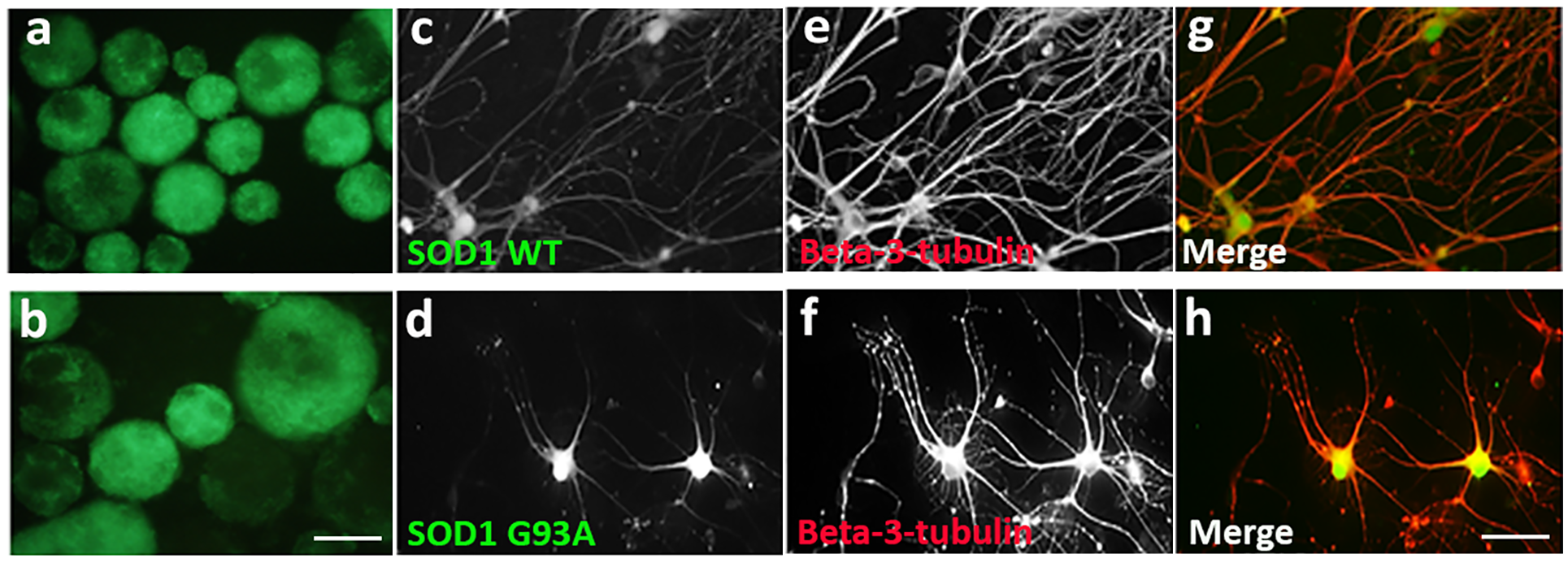

Supplement: Supplementary file 2 — High Resolution Image (TIF 3969 kb) [file 13311_2016_505_MOESM1_ESM.tif]

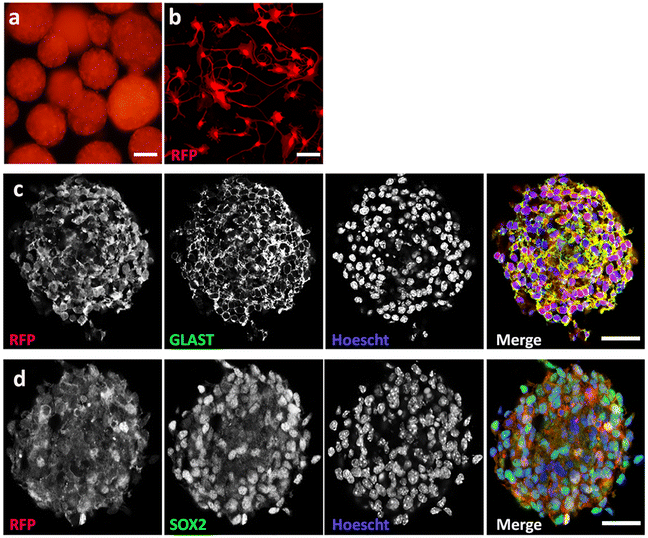

Supplement: Supplementary file 3 — Overview of RFP-expressing bNCSC neurospheres in pre-differentiation (a) and the bNCSC monolayer during differentiation stage (b). Immunostaining of bNCSC neurospheres shows expression of the glial specific marker GLAST (c) and the neural crest stem cell specific markers SOX2 (d). Scale bars: a,b 20 μm; c,d 50 μm. (GIF 164 kb) [file 13311_2016_505_Fig8_ESM.gif]

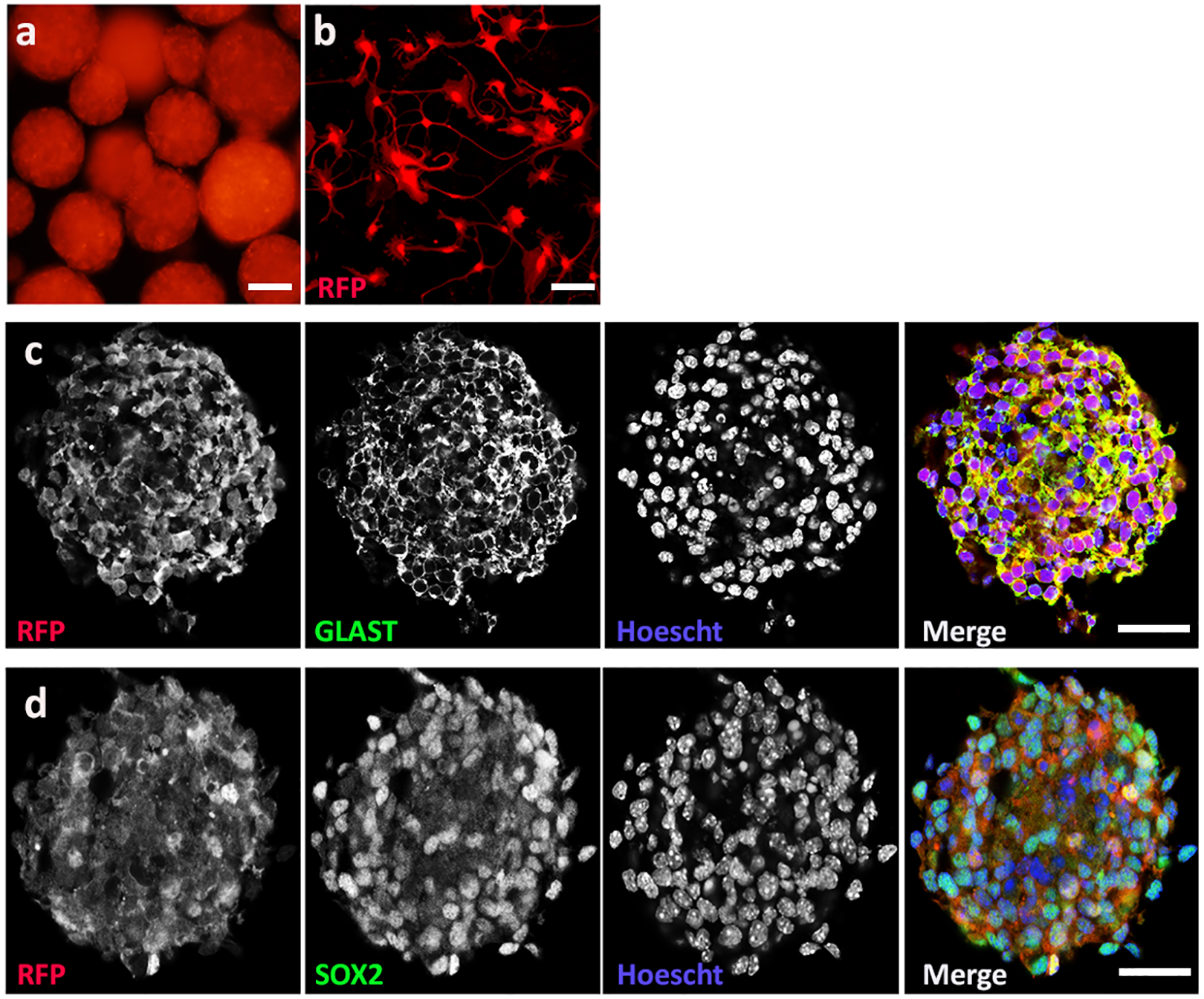

Supplement: Supplementary file 4 — High Resolution Image (TIF 6408 kb) [file 13311_2016_505_MOESM2_ESM.tif]

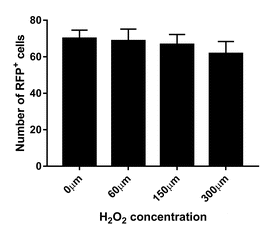

Supplement: Supplementary file 5 — Effect of hydrogen peroxide (H2O2) on the survival of bNCSCs in vitro. Cultures were exposed to different concentrations of H2O2 for 3 hours. The survival of bNCSCs is not affected by different concentrations of H2O2 (GIF 5 kb) [file 13311_2016_505_Fig9_ESM.gif]

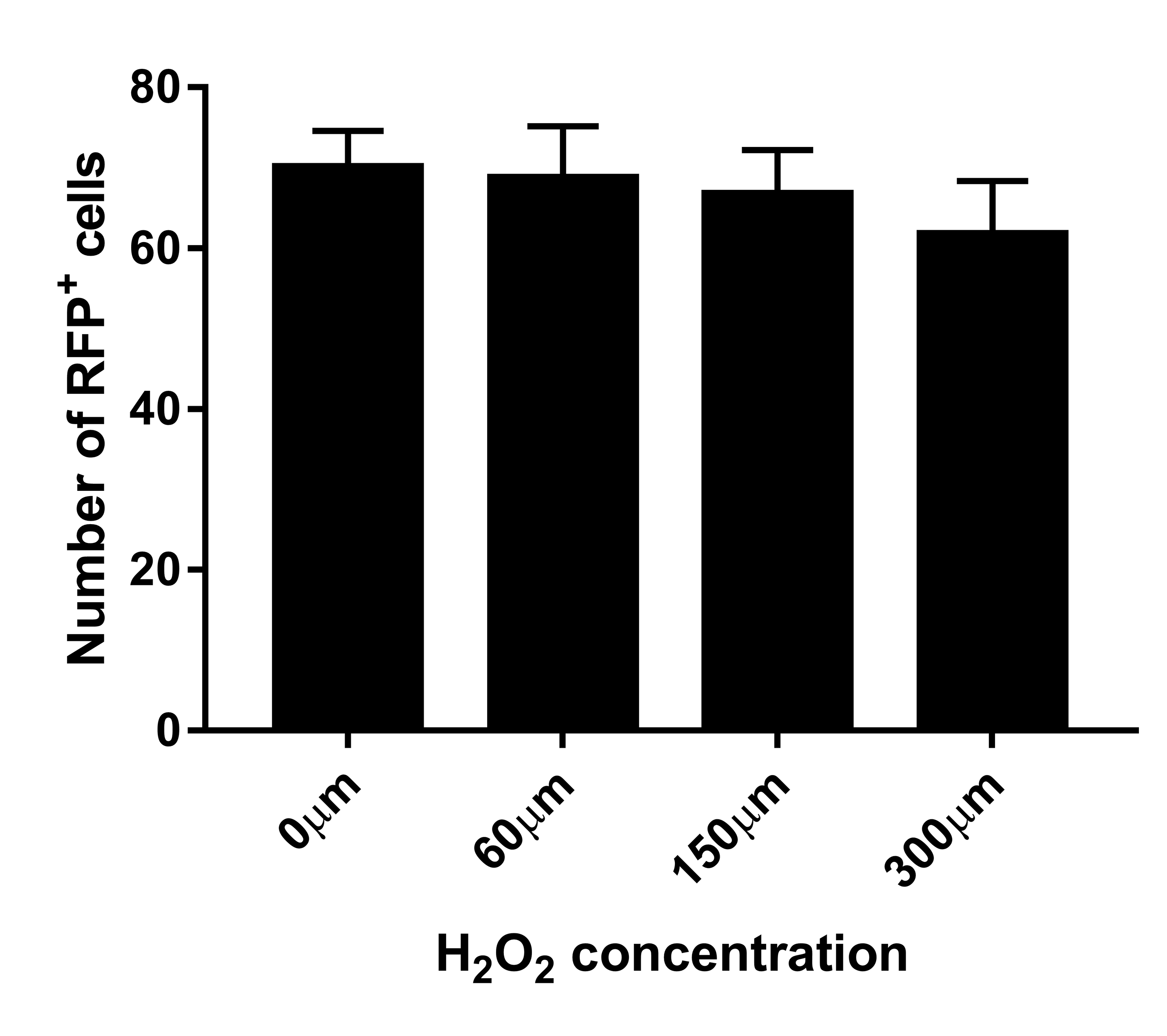

Supplement: Supplementary file 6 — High Resolution Image (TIF 18434 kb) [file 13311_2016_505_MOESM3_ESM.tif]

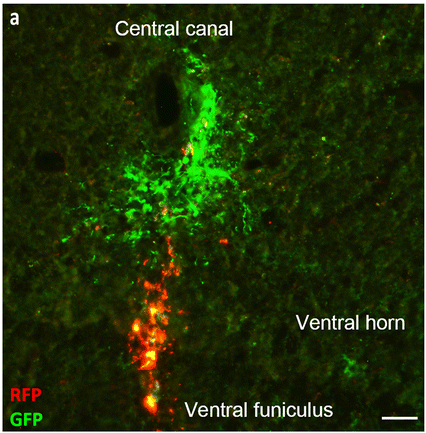

Supplement: Supplementary file 7 — bNCSCs and SOD1G93A MNs one week after implantation to the ventral horn. SOD1G93A MNs (green) are located in the grey matter close to the central canal and together with bNCSCs (red) in the white matter of the ventral funiculus. Scale bar: 50 μm (GIF 127 kb) [file 13311_2016_505_Fig10_ESM.gif]

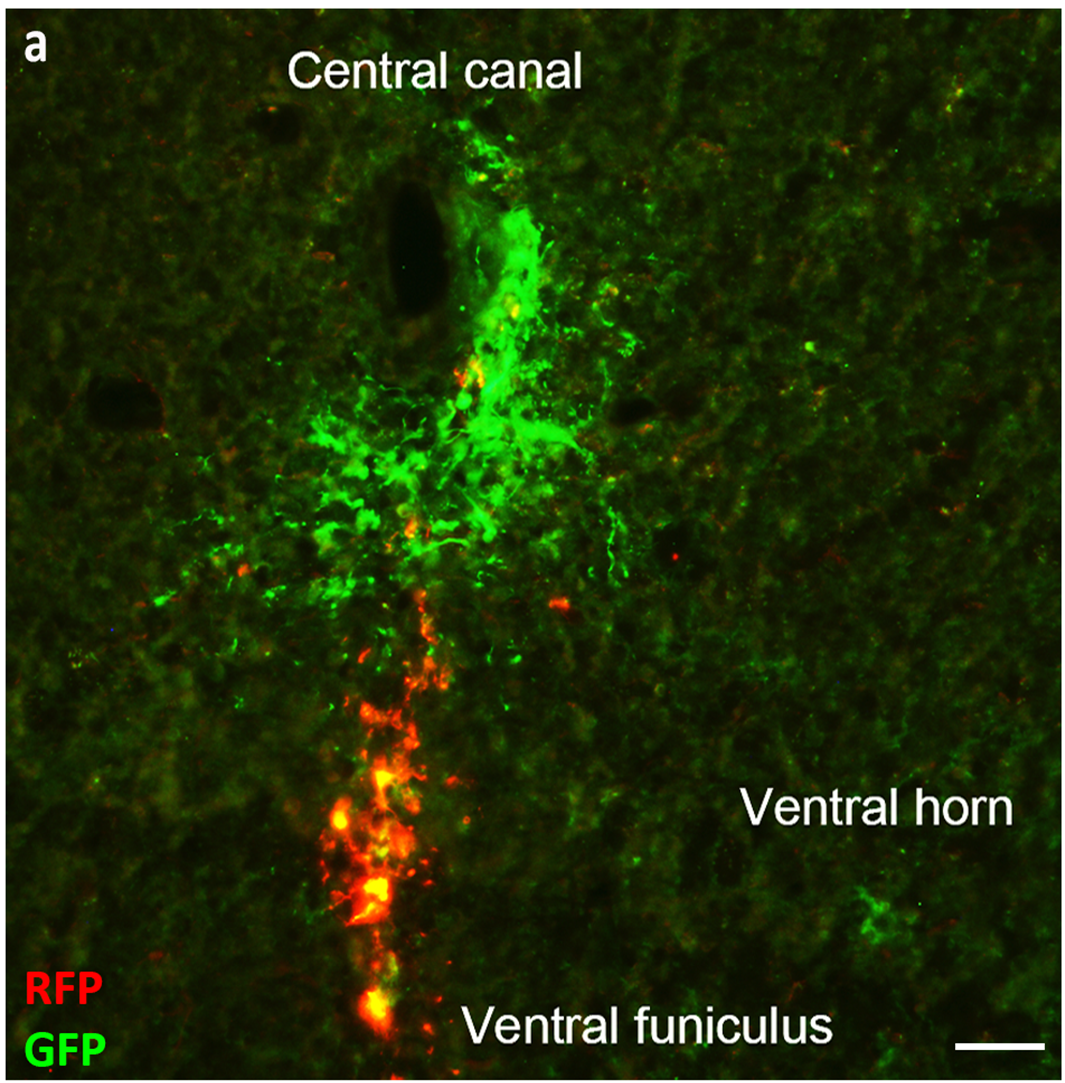

Supplement: Supplementary file 8 — High Resolution Image (TIF 3407 kb) [file 13311_2016_505_MOESM4_ESM.tif]
